# Polyphenol derivatives – potential regulators of neutrophil activity

**DOI:** 10.2478/v10102-012-0011-8

**Published:** 2012-06

**Authors:** Katarína Drábiková, Tomáš Perečko, Radomír Nosáľ, Juraj Harmatha, Jan Šmidrkal, Viera Jančinová

**Affiliations:** 1 Institute of Experimental Pharmacology & Toxicology, Slovak Academy of Sciences, SK-84104 Bratislava, Slovakia; 2 Institute of Organic Chemistry and Biochemistry, Academy of Sciences of the Czech Republic, v.v.i., Flemingovo namesti 2, 166 10, Praha, Czech Republic; 3 Institute of Chemical Technology Prague, Technická 5, 166 28 Praha 6-Dejvice, Czech Republic

**Keywords:** activity of neutrophils, reactive oxygen species, natural polyphenols

## Abstract

The study provides new information on the effect of natural polyphenols (derivatives of stilbene – resveratrol, pterostilbene, pinosylvin and piceatannol and derivatives of ferulic acid – curcumin, N-feruloylserotonin) on the activity of human neutrophils in influencing oxidative burst. All the polyphenols tested were found to reduce markedly the production of reactive oxygen species released by human neutrophils on extra-and intracellular levels as well as in cell free system. Moreover, pinosylvin, curcumin, N-feruloylserotonin and resveratrol decreased protein kinase C activity involved in neutrophil signalling and reactive oxygen species production. Our results suggest that due to their anti-neutrophil activity, the polyphenols tested might be attractive candidates in therapeutic development.

## Introduction

Numerous studies reported polyphenols as potential therapeutic agents against inflammatory diseases including obesity, diabetes, cardiovascular and neurodegenerative diseases, rheumatoid arthritis, cancer and aging (Stevenson & Hurst, 2007; Pandey & Rizvi, 2009; Obrenovich *et al.,* 2010; Štefek, 2011). Several mechanisms of the anti-inflammatory effect of polyphenols have been proposed, yet information about their favourable effects on neutrophils is rare. Activated neutrophils release large amounts of enzymes and reactive oxygen species (ROS) to the extracellular milieu, overpowering the local antioxidant defense systems and contributing to tissue damage and the amplification of the inflammatory process. In the physiopathology of many inflammatory diseases, the involvement of ROS produced by neutrophils has been attracting interest in the discovery of new compounds with antioxidant and immunomodulatory properties, which might modulate neutrophil activity.

**Figure 1:**
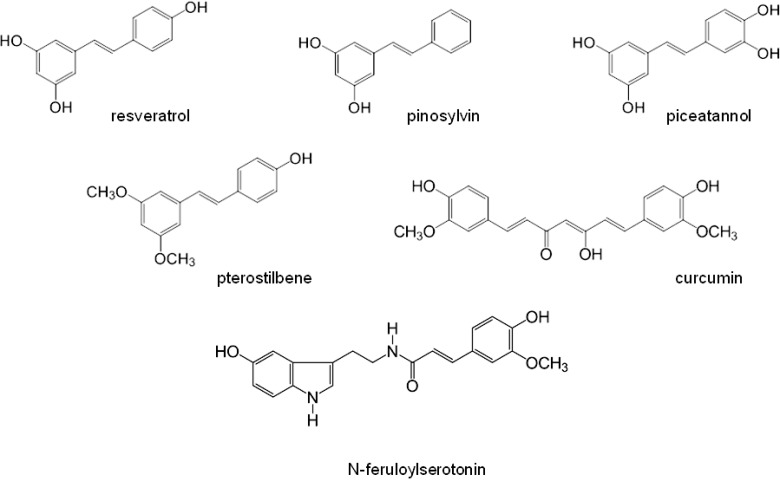
Structure of resveratrol, pinosylvin, piceatannol, pterostilbene, curcumin and N-feruloylserotonin.

In this context, we investigated the effect of some compounds of natural origin (derivatives of stilbene, resveratrol, pinosylvin, pterostilbene, piceatannol and derivatives of ferulic acid – curcumin, N-feruloylserotonin, Figure 1) on the activity of human neutrophils *in vitro* with respect to their influence on oxidative burst. Here we summarize the results obtained.

## Effect on extra- and intracellular ROS generation

For activation of human isolated neutrophils we used the soluble stimulus phorbol 12-myristate 13-acetate (PMA) which stimulates ROS generation by direct activation of protein kinase C (PKC). PMA is useful in investigating signal transduction pathways leading to NADPH-oxidase activation in plasma (extracellular – potentially dangerous for host tissues) and granule membranes (intracellular – involved in elimination of phagocytosed pathogens and fulfilling a regulatory role) (Karsson, 2002). Neutrophil activation was evaluated by using luminol/isoluminol-amplified chemiluminescence (CL), which allows to differentiate the effect of polyphenols on extracellular and intracellular oxidant production (Jančinová *et al.,* 2006). Table 1 shows the effects of the substances tested on extracellular CL of isolated human neutrophils stimulated with PMA (0.05 µmol/l) in the 0.01–100 µmol/l concentration scale. The most effective concentration tested was 100 µmol/l of all substances, reaching 99% inhibition for curcumin, piceatannol, resveratrol and N-feruloylserotonin, and 87% for pinosylvin and pterostilbene. The effective rank order of the substances tested producing 50% inhibition of control extracellular CL of neutrophils is: resveratrol > N-feruloylserotonin ≥ curcumin > piceatannol > pterostilbene > pinosylvin (Table 1) (Perečko *et al.,* 2008; Jančinová *et al.,* 2009, 2011; Nosáľ *et al.,* 2010Nosáľ *et al.,* 2011).

**Table 1:** Effect of curcumin, N-feruloylserotonin, resveratrol, piceatannol, pterostilbene and pinosylvin on extracellular neutrophil chemiluminescence stimulated with PMA.

Inhibition of extracellular chemiluminescence (%)						
µmol/l	Curcumin	N-feruloyl serotonin	Resveratrol	Piceatannol	Pterostilbene	Pinosylvin
0.01	9.9±3.8	4.0±1.0	17.4±2.6	6.0±2.8	20.6±3.4	–1.6±1.2
0.1	15.7±3.8	1.8±2.3	22.2±3.7	9.7±3.8	19.8±4.5	1.2±3.1
1	31.0±1.9	30.7±2.6	44.3±3.0	23.0±3.5	31.0±2.8	9.4±2.8
10	86.1±2.3	91.1±0.8	88.6±1.4	92.5±1.2	70.9±5.8	39.6±2.1
100	99.7±0.0	99.6±0.1	99.5±0.1	100±0	88.2±3.4	86.2±1.0
**IC**_**50**_	**1.84**	**1.82**	**0.85**	**1.87**	**2.25**	**15.07**

Percentage inhibition was calculated on the basis of integrated values of chemiluminescence (CL) over 1800s. Mean ± SEM, n=6–8.

IC_50_ – doses producing 50% inhibition of control extracellular CL.

In the intracellular milieu of human neutrophils, no effects of the substances tested were observed at concentrations 0.01–1 µmol/l. However, the effects differed at concentrations 10 µmol/l, the inhibitory effect of resveratrol, pinosylvin and curcumin increased rapidly, whereas pterostilbene, piceatannol and N-feruloylserotonin were less operative. In the concentration of 100 µmol/l all compounds tested (except pterostilbene) caused at least 90% inhibition of intracellular CL (Perečko *et al.,* 2008; Jančinová *et al.,* 2009, 2011; Nosáľ *et al.,* 2010, 2011). The effective rank order of the substances tested producing 50% inhibition of control intracellular CL of neutrophils is: curcumin > pinosylvin > resveratrol > N-feruloylserotonin > piceatannol > pterostilbene (Table 2).

**Table 2:** Effect of curcumin, N-feruloylserotonin, resveratrol, piceatannol, pterostilbene and pinosylvin on intracellular neutrophil chemiluminescence stimulated with PMA.

Inhibition of intracellular chemiluminescence (%)						
µmol/l	Curcumin	N-feruloyl serotonin	Resveratrol	Piceatannol	Pterostilbene	Pinosylvin
0.01	–8.2±4.6	–1.1±3.2	–0.2±1.7	–1.7±2.6	–3.5±2.8	2.1±3.2
0.1	–6.9±5.5	–1.1±2.2	–3.6±2.8	–2.9±1.4	–2.5±1.1	0.1±3.0
1	8.1±6.1	7.9±4.6	4.9±1.6	–0.2±1.3	3.9±1.9	8.4±5.2
10	81.4±2.4	38.9±4.7	71.4±3.2	39.6±2.1	39.5±3.4	66.0±4.3
100	93.9±1.4	90.0±1.0	96.4±0.7	99.3±0.1	69.3±5.5	90.5±1.4
**IC**_**50**_	**3.57**	**8.40**	**6.19**	**13.17**	**21.58**	**4.45**

Percentage inhibition was calculated on the basis of integrated values of chemiluminescence (CL) over 1800s. Mean ± SEM, n=6–8.

IC_50_ – doses producing 50% inhibition of control intracellular CL.

## Effect on protein kinase C activity

Polyphenols have been suggested to affect cell function by modifying plasma membrane structure and physical characteristics such as fluidity and electrical properties. These effects can be observed both when polyphenols are adsorbed on the membrane (polyphenols could provide a physical barrier for hydrosoluble radicals) and when they are inserted into the bilayer (polyphenols would be in close proximity so as to scavenge lipid soluble radicals) (Fraga *et al.,* 2010). In biological systems, ROS are generated by a number of enzymatic systems and the modifications of plasma membrane structure can result in functional changes including the activity of membrane-associated enzymes, ligand-receptor interactions, ion and/or metabolite fluxes, and the modulation of signal transduction (Khlebnikov *et al.,* 2007). Stimulation of neutrophils with PMA is accompanied by increased phosphorylation of protein kinase C (PKC) isoenzymes α and β II, which directly participate in the activation of neutrophil NADPH oxidase (Fontayne *et al.,* 2002; Klink *et al.,* 2009). On the other hand, inhibition of PKC or down-regulation of its intracellular expression and activity has also been proposed as an important mechanism of polyphenol antioxidant effect (Khlebnikov *et al.,* 2006). Moreover, evidence has been increasing on the selective inhibition of PKC beneficially applied in a new therapeutic strategy for treating diseases related to oxidative stress (Lee *et al.,* 2009).

**Figure 2:**
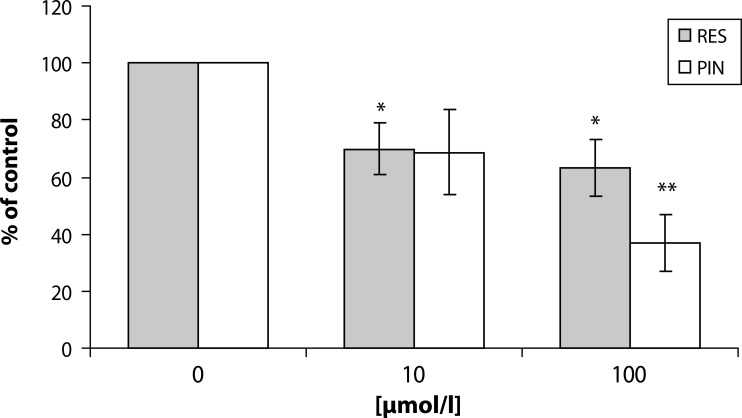
PKC phosphorylation in PMA stimulated human neutrophils treated with 10 and 100 µmol/l resveratrol (RES) and pinosylvin (PIN). The values of phosphorylation are presented as percentage of stimulated (PMA) control. Phosphorylated PKC isoenzymes (α and βII) were isolated by Western blotting and detected by Phospho-PKC α/βII (Thr638/641) Antibody. Mean ± SEM, n=4. **p<0.01, *p<*0.05 as compared with the control in the absence of the substances tested.

**Figure 3:**
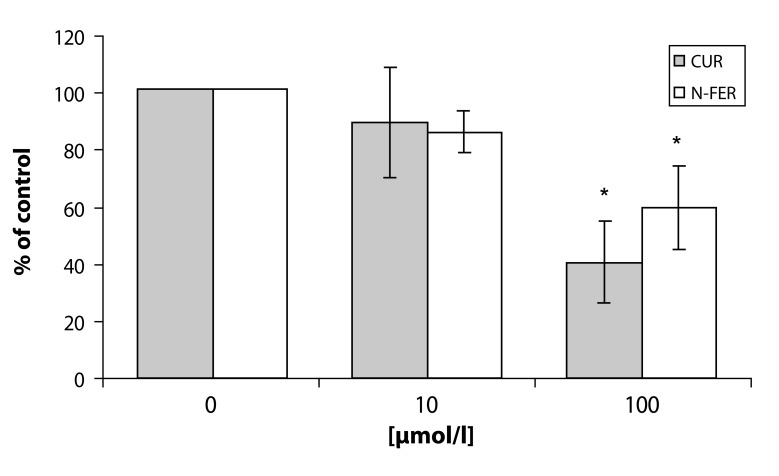
PKC phosphorylation in PMA stimulated human neutrophils treated with 10 and 100 µmol/l curcumin (CUR) and N-feruloylserotonin (NFER). The values of phosphorylation are presented as percentage of stimulated (PMA) control. Phosphorylated PKC isoenzymes (α and βII) were isolated by Western blotting and detected by Phospho-PKC α/βII (Thr638/641) Antibody. Mean ± SEM, n=4. *p<0.05 as compared with the control in the absence of the substances tested.

In the attempt to elucidate the molecular mechanisms involved in the reduction of ROS production by human neutrophils, we examined the effect of resveratrol, pinosylvin, pterostilbene, piceatannol, curcumin and N-feruloylserotonin on the phosphorylation of PKC α/βII (Thr638/641). Pterostilbene and piceatannol did not influence PKCα/βII phosphorylation after PMA stimulation, and this effect could be explained by low accessibility of these compounds to the cell compartments containing the enzyme. However resveratrol, pinosylvin, curcumin and N-feruloylserotonin in the concentrations of 10 and 100 µmol/l effectively reduced PKCα/βII phosphorylation (Figures 2, 3) (Jančinová *et al.,* 2009; Perečko *et al.,* 2010; Nosáľ *et al.,* 2011). As described for resveratrol (Slater *et al.,* 2003), the inhibitory effect on PKC activity might result from competition between polyphenols and phorbol ester for binding to the C1 domains of the enzyme, or from the conformational change in the membrane-associated enzyme. Moreover, studies of docking simulation into PKC showed efficient inhibition of PKC by polyphenols (Račková *et al.,* 2009). The structure-dependence of the inhibitory effect of polyphenolic antioxidants on signal transduction enzymes, such as PKC, has been suggested also by Ursini *et al.* (1994) and Varga *et al.* (2006).

## Effect on cell free system

We investigated the participation of direct antioxidant activity of polyphenols in decreasing peroxyl radical formation in a cell free CL system consisting of luminol, horseradish peroxidase and hydrogen peroxide. The luminol reaction is highly dependent on the participation of myeloperoxidase, thus the reduction of the CL signal might be the result of decreased availability of peroxidase, due either to its decrease of activity or liberation from azurophilic granules of neutrophils. The possible interaction of polyphenolic antioxidants with peroxidase is supported by findings of Franck *et al.* (2008) who demonstrated the interaction of curcuminoids with the active site of myeloperoxidase. The inhibitory effect of pinosylvin and pterostilbene on MPO release was described by Pečivová *et al.* (2010). Our results showed that the observed reduction of oxidants, produced by neutrophils extra- and intracellularly, may involve antioxidant activity of the polyphenols tested, as manifested by the effective inhibition of CL generated by cell free system (Table 3). The effective rank order of the substances tested producing 50% inhibition of cell free CL is: piceatannol > resveratrol ≥ pterostilbene = N-feruloylserotonin > pinosylvin (Table 3).

**Table 3:** Effect of N-feruloylserotonin, resveratrol, piceatannol, pterostilbene and pinosylvin on cell free chemiluminescence.

Inhibition of cell-free chemiluminescence (%)					
µmol/l	N-feruloyl serotonin	Resveratrol	Piceatannol	Pterostilbene	Pinosylvin
0.01	0.8±1.0	0.3±0.7	0.6±1.2	–0.3±0.4	–0.4±1.3
0.1	3.6±1.2	3.8±1.6	6.2±0.6	–1.4±0.6	0.1±0.9
1	25.5±0.8	31.6±0.7	66.4±0.1	12.8±0.0	5.4±0.8
10	97.8±0.1	98.1±0.0	99.3±0.0	76.6±0.5	62.7±0.5
100	98.8±0.1	99.4±0.1	99.3±0.0	99.2±0.0	99.1±01.1
**IC**_**50**_	**1.70**	**1.50**	**0.60**	**1.70**	**6.60**

Percentage inhibition was calculated on the basis of integrated values of chemiluminescence (CL) over 600s. Mean ± SEM, n=3.

IC_50_ – doses producing 50% inhibition of control cell free CL system.

The mechanisms involved in antioxidant activity of polyphenols are complex, related to the structure of the compound. Stilbenes are naturally occurring more in Z form, which is also more effective compared to the E form (Aggarwal *et al.,* 2004; Šmidrkal *et al.,* 2010). The molecule has a common C6–C2–C6 structure, consisting of two aromatic rings linked through a two-carbon bridge with a double bond. Depending on the character of the substituent, the phenols in stilbene could be either saturated or drawn off with electrons. This may influence electron donor/acceptor properties of stilbene derivatives and thus their antioxidant activity.

**Resveratrol** is a phytoalexin structurally related to stilbenes. Resveratrol has been an effective scavenger of hydroxyl, superoxide, and metal induced radicals. In cells producing ROS, its antioxidant abilities have also been documented (Rizvi & Pandey, 2010). The authors further showed its protective effect against lipid peroxidation in cell membranes. The three hydroxyl groups of resveratrol were found to participate in an extensive three-dimensional hydrogen-bonding network. The hydrogen bonding due to the molecular packing in the crystal structure demonstrates the ready mobility of up to three hydrogen atoms per resveratrol molecule (Rizvi & Pandey, 2010).

Removal of the hydroxyl group from resveratrol in position 4′ results in **pinosylvin**. By this change, pinosylvin (partition coefficient-logP: 3.8) is more lipophilic than resveratrol (logP: 3.1). Both pinosylvin hydroxyl groups are located in *meta* position (with respect to the ethylene bridge of the stilbene molecule), i.e. in an arrangement less favourable both for electron abstraction and for the distribution of the unpaired electron (Fan *et al.,* 2009; Queiroz *et al.,* 2009).

In our experiments with human neutrophils, both pinosylvin and resveratrol at the concentrations 10 and 100 µmol/l, were effective in reducing intracellular ROS production and PKCα/βII phosphorylation (Table 2, Figure 2). Thus, we suggest that the removal of 4′-OH does not affect either intracellular antioxidant activity or PKCα/βII phosphorylation. In agreement with other studies (Stojanovič *et al.,* 2001; Roupe *et al.,* 2006), we found that the 4′-OH group in the structure of resveratrol was crucial for a strong extracellular antioxidant effect (Perečko *et al.,* 2008). The less potent antioxidant effect of pinosylvin in comparison with resveratrol was established also in the cell free CL system (Table 3).

The change in resveratrol, i.e. methoxylation in 3,5 – position, leads to **pterostilbene** (logP 4.1). The peroxyl radical scavenging activity of pterostilbene appears to be similar to that of resveratrol. The antioxidant activity of pterostilbene was first demonstrated in vitro by its inhibition of methyl linoleate oxidation. Pterostilbene was reported to scavenge 1,1-diphenyl-2-picryl-hydrazyl (DPPH) free radicals, 2,2′azo-bis(2amidinopropane) (ABAP) derived peroxyl radicals and to inhibit the oxidation of citronellal, as well as lipid peroxidation in rat liver microsomes and in cultured human fibroblasts (Roupe *et al.,* 2006; Pan *et al.,* 2008).

Substitution of two hydroxyl groups with methoxy groups increased the lipophilicity parameter R_M_ (retention constant*)* of resveratrol from 0.21 to 1.45 which applies for pterostilbene. This may enhance the bioavailability of pterostilbene in contrast to the low bioavailability of resveratrol. On the other side, lipophilicity may influence the transition of substances through the cell membrane into cytosol. Also the number and position of hydroxyl groups play a role in the antioxidant effects of polyphenols in different cell systems. Considering the effects of different resveratrol derivatives on the production of thiobarbituric acid reactive substances (TBARS) in normal human fibroblasts, pterostilbene was as good as resveratrol. Its 4′-methoxy derivative as well as 3, 4′, 5-trimethoxystilbene did not exert a significant inhibition of TBARS production (Stivala *et al.,* 2001). These results support our findings with chemiluminescence assay in whole blood. The 3, 5-methoxy groups increased the antioxidant properties of pterostilbene compared to resveratrol in whole human blood (Perečko *et al.,* 2008). In the extracellular space of isolated neutrophils, we found that pterostilbene at the concentration 10 µmol/l was less effective than resveratrol (Table 1). Despite the highest lipophilicity among the substances tested, pterostilbene was the least effective against intracellular CL of isolated neutrophils. This may be due to the requirement of free 3,5-OH groups in intracellular activity. The results are indicating that 3,5-meta-methoxyl groups decrease the extracellular and especially the intracellular activity of pterostilbene compared to resveratrol. After PMA stimulation, pterostilbene in either concentration used (10 and 100 µmol/l), failed to induce significant changes in PKC α/β II (Thr638/641) phosphorylation. Thus logP is not the only condition operative in intracellular activity (Perečko *et al.,* 2008Perečko *et al.,* 2010).

Due to the structural similarities between **piceatannol** and resveratrol, it has been hypothesised that piceatannol may also possess potent antioxidant activity. Piceatannol was shown to be a more effective scavenger of nitric oxide and hydrogen peroxide compared to resveratrol. The additional hydroxyl group of piceatannol makes it more reactive compared to resveratrol. In our experiments, piceatannol exerted a more intensive inhibition of chemiluminescence in free cell system in comparison to resveratrol, yet in the inhibition of extra-and intracellular ROS production resveratrol was more effective (Tables 1,2,3) (Jančinová *et al.,* 2011). The mechanism by which the naturally occurring polyphenolic compound resveratrol and its metabolite piceatannol scavenge free radicals was studied using experimental and density functional theory methods (Rossi *et al.,* 2008). Piceatannol was found to be more efficient than resveratrol because (i) by sharing its 3′-OH hydrogen atom with its adjacent neighbour, O-4′, the abstraction and transfer of the 4′-H atom to the free radical becomes easier, and (ii) the resulting piceatannol semiquinone radical is more stable. The interaction of both resveratrol and piceatannol with model membranes composed of phosphatidylcholine (DMPC and DPPC) was investigated by means of fluorescence spectroscopy, differential scanning calorimetry and electron spin resonance spectroscopy pointing to the preferential interaction of resveratrol and piceatannol with the headgroup region of lipid bilayer (Wesołowska *et al.,* 2009).

**Curcumin** was found to be an effective antioxidant in different *in vitro* assays including: 2,2-diphenyl-1-picryhydrazyl (DPPH), 2,2’-azino-bis(3-ethylbenzothiazoline-6-sulphonic acid) (ABTS), and N,N-dimethyl-p-phenylenediamine (DMPD). The antioxidant activity of curcumin results from the presence of phenolic and central methylene hydrogens in its molecule (Ak & Gülçin, 2008; Lin *et al.,* 2008). In our experiments, curcumin reduced dose-dependently oxidant formation in human neutrophils at extra- and intracellular level and in cell free system, and it effectively reduced protein kinase C activation (Jančinová *et al.,* 2009). This is consistent with the results found by Franck *et al.* (2008), Deby-Dupont *et al.* (2005), in which curcumin inhibited ROS generation of neutrophils and lymphocytes stimulated with PMA, as well as the fibroblast PKC. The antioxidant properties of curcumin are based on its lipid peroxidation lowering effects through the ability to maintain the cellular status of antioxidant enzymes, like superoxide dismutase, catalase and glutathione peroxidase. Indeed, curcumin has been shown to increase reduced glutathione (GSH) levels, which leads to lowered ROS production (Rahman *et al.,* 2006; Rahman, 2008). When curcumin was compared to other antioxidants in a lipid peroxidation assay of linoleic acid, it inhibited the lipid peroxidation by 97.3% as compared to standard antioxidants: 84.6% for α-tocopherol and 95.6% for trolox (Ak & Gülçin, 2008). In the micro to millimolar range, curcumin was shown to scavenge ROS, i.e. superoxide anion, hydrogen peroxide and nitric oxide (NO), both *in vitro* and *in vivo* (Obrenovich *et al.,* 2010). Curcumin between 1 and 50 mmol/l, scavenged ROS as determined by electron pulse resonance spectroscopy and it was much faster in terms of quenching ROS than other polyphenols (resveratrol and quercetin).

**N-feruloylserotonin** conjugate was identified as the major and unique phenolic constituent of defatted safflower seeds. We found that N-feruloylserotonin markedly diminished oxidant formation in cell free CL system as well as in human neutrophils, both at extra- and intracellular level. Our further results suggest that one of the molecular effects of N-feruloylserotonin might involve the inhibition of PKCα/βII activity (Nosáľ *et al.,* 2011). N-feruloylserotonin was demonstrated to exert an inhibitory effect on overproduction of mitochondrial superoxide by acting as scavenger of the superoxide, and its ROS-scavenging activity was comparable with that exerted by 40 µmol/l α-tocopherol (Piga *et al.,* 2009). The antioxidant effect of N-feruloylserotonin was shown to be dependent on its structure (Piga *et al.,* 2009; Takahashi & Miyazawa, 2011). Compared to serotonin, the authors reported a higher ability of N-feruloylserotonin to reduce ROS, suggesting a strong effect of the serotonin and ferulic acid moieties and their amide linkage on the antioxidant activity of N-feruloylserotonin.

## Conclusion

The presented findings indicate that the derivatives of stilbene – resveratrol, pterostilbene, pinosylvin and piceatannol and the derivatives of ferulic acid – curcumin, N-feruloylserotonin may be suitable inhibitors of neutrophil activation, implying their anti-inflammatory potential.
